# Resilience and Mental Health in the Polish Population during the COVID-19 Lockdown: A Mediation Analysis

**DOI:** 10.3390/jcm10214974

**Published:** 2021-10-26

**Authors:** Janusz Surzykiewicz, Karol Konaszewski, Sebastian Skalski, Paweł Piotr Dobrakowski, Jolanta Muszyńska

**Affiliations:** 1 Faculty of Philosophy and Education, Catholic University of Eichstaett-Ingolstadt, 85051 Eichstaett, Germany; 2 Faculty of Education, Cardinal Wyszynski University in Warsaw, 01815 Warsaw, Poland; 3 Faculty of Education, University of Bialystok, 15328 Bialystok, Poland; k.konaszewski@uwb.edu.pl (K.K.); jolamusz@uwb.edu.pl (J.M.); 4 Institute of Psychology, Polish Academy of Sciences, 00950 Warsaw, Poland; sebastian.skalski@sd.psych.pan.pl; 5 Institute of Psychology, Humanitas University, 41200 Sosnowiec, Poland; paweldobrakowski@interia.pl

**Keywords:** resilience, mental health, well-being, anxiety about COVID-19, obsession with COVID-19, stress over COVID-19

## Abstract

The aim of this paper was to assess the state of resilience and well-being in the Polish population during the COVID-19 pandemic. We also assessed the relationship between resilience and mental health. Finally, we tested the mediating role of COVID-19 anxiety, persistent thinking, and the stress burden in the relationship between mental health and resilience. This research perspective can provide important insights into how individuals can become mentally stronger during the COVID-19 pandemic. Methods: This study included 1758 people (73% women) aged 18–80 years. The procedure consisted of completing a questionnaire measuring well-being, COVID-19 anxiety, obsession with COVID-19, stress over COVID-19, and resilience. Results: Bootstrap sampling analysis showed significant partial mediators for the relationship between resilience and well-being. Important mediators were coronavirus anxiety, persistent thinking, and perceived stress. Conclusions: The results of the present study clearly indicate that resilience as a protective factor is associated with reduced anxiety about COVID-19, perceived stress burden, obsessive thoughts about the pandemic, and increased well-being of individuals. Resilience plays an important role in minimizing negative and enhancing positive health indicators in the face of challenging life events.

## 1. Introduction

Anxiety, a sense of being burdened, and stress are common responses to perceived or real threats, and also when faced with uncertainty or the unknown. So, it is understandable that people feel such tension in the context of the COVID-19 pandemic [1,2,3,4]. In addition to the fear of contracting the virus, there are significant changes in everyday life, as individuals are restricted in their functioning as part of efforts to contain and slow down the spread of the virus. In the face of this new reality, the World Health Organization (WHO) and researchers are paying attention to the need for a two-dimensional approach and recommend psychological interventions aimed at minimizing negative indicators of mental health (e.g., anxiety, depression, stress) and activating positive indicators (e.g., well-being, quality, and satisfaction with life) [5,6,7]. Therefore, the current definitions of health deviate from a one-sided understanding of disease and focus not only on reducing or eliminating disease manifestations, but also on taking into account pro-health aspects, considering physical, mental, and social areas. In this respect, a two-dimensional model of mental health is a solid basis for holistic and accurate diagnosis of individual health-related indicators [5,8]. In this paper, it was decided to use both types of mental health indicators. According to the assumptions of psychological resilience theories [9,10,11,12,13], in addition to both types of mental health indicators, personal resources and assets should be identified as means of helping individuals overcome difficulties and adapt to circumstances. For these reasons, this study looked at the role that resilience plays in mental health in the general population, considering both positive (well-being) and negative (anxiety, obsessive thinking, burden of stress) indicators.

The COVID-19 pandemic has created a critical global situation, with a particularly severe impact on the quality of life and mental health of many people. Publications on mental health during the pandemic and related determinants are still increasing. Many of them report worrying consequences for psycho-emotional and social functioning or increased susceptibility to mental health problems (e.g., high risk of depression, stress, and more frequent suicidal thoughts and behavior) [14]. According to recent meta-analyses, the incidence of stress, anxiety, and depression in the general population as a result of a pandemic is around 30% [15,16,17]. The current pandemic has been accompanied by numerous stressors that lead to anxiety and hopelessness [1,15,18]. 

COVID-19 research and media reports have revealed a rise in fear related to contracting the virus. Though fear is a common psychological outcome during a pandemic, the current pandemic is a continuously evolving disease outbreak and has unique risk factors. Therefore, fear related to COVID-19 could manifest in not only fear and anxiety related to contracting the disease and dying, but also associated socio-occupational stress [19,20]. The data also show that people who are isolated and quarantined have higher levels of anger, confusion, and post-traumatic stress [2]. The epidemic experience increases the long-term level of depression [21,22,23] and may also increase future suicide rates [24]. Experiencing more unpleasant events in life and having difficulty coping with them are also predictors of anxiety, stress, and depression [25]. Research indicates that the COVID-19 pandemic has caused significant declines in mental health, life satisfaction, and well-being in China, the USA, Japan, Germany, Ireland, and New Zealand, among countries. Therefore, we expect that the COVID-19 pandemic will also have a negative impact on mental health in Poland. In such a situation, it seems necessary to pay attention to resources on immunity, which are the basis for, inter alia, the theory of salutogenesis by Antonovsky [26] and the theory of resilience [9,11,27], which may protect against the negative impact of a pandemic and be helpful in finding answers to questions about maintaining and strengthening positive aspects of health [28,29]. 

Dealing with a pandemic requires an adequate ability to withstand failure, adapt positively, and react to adversity. It also requires the ability to cope with significant changes and take responsibility by recovering from adversity, uncertainty, and negativity, and even to make positive changes. This ability is referred to as resilience [30], which can be developed and strengthened and change over time. Strengthening mental health and developing specific facilities and skills can be an opportunity to develop the well-being of individuals.

It has been shown that some people are more mentally resistant to adversity than others, and that patterns of vulnerability and resilience differ [31]. The literature consistently shows a negative relationship between resilience and anxiety, persistent thinking, depression, and psychological distress, especially in the case of natural disasters such as the 2010 Haiti earthquake [32] and Hurricane Katrina in 2005 [33]. It is therefore crucial for researchers and clinicians to identify the factors that help build up the mental health of individuals and the well-being of society. It should be noted that, according to recent reports, resilience is a significant predictor of mental health behavior during the COVID-19 pandemic [34]. Researchers indicate that resilience may, inter alia, reduce the symptoms of post-traumatic stress, anxiety about the coronavirus, and the intensification of complicated grief in people who could not take care of their relatives’ final arrangements during the lockdown [35,36]. Researchers also found that the average immunity in the population during the outbreak was lower than published standards, but was higher among those who went outside more often, exercised more, had greater social support from family, friends, and other important people, slept better, and prayed more [37,38]. It should therefore be noted that resilience in the face of a pandemic is associated with modifiable factors. However, the impact of resilience on mental health (WHO) in the Polish population in the context of the COVID-19 pandemic has not been well documented. 

Previous reports on the spread of infectious diseases found clear associations between perceived anxiety and stress over a pandemic and depressed well-being and symptoms of PTSD [39,40,41]. It therefore seems that perceived anxiety and stress may be effective markers for mental functioning during COVID-19. Accordingly, in recent reports, anxiety over coronavirus explained the impact of resilience to PTSD symptoms [42]. A similar mediator in the relationship between resilience and PTSD was reported to be persistent thinking about COVID-19 [43]. Persistent thinking is associated with passive attention to negative emotions [44]. According to Beck, distortions in information processing lead to a preoccupation with the threat and an underestimation of the ability to deal with the threat, which ultimately causes pathological anxiety or distress [45,46]. In the case of perceived stress, no similar analyses have been conducted so far. However, Gustafsson and colleagues [47] showed that perceived stress fully mediates the relationship between optimism and burnout symptoms, and according to Wang and colleagues [48], perceived stress partially mediates the relationship between hope and PTSD symptoms. Therefore, it seems that the direction of the relationship between resilience and well-being may be explained by anxiety over coronavirus, persistent thinking about COVID-19, and perceived stress.

The pandemic, while also being a global public health threat with multifaceted serious consequences for people’s lives and mental health, is raising the awareness not only of researchers, but also of practitioners and politicians around the world regarding the urgent need to focus on resilience resource-based programs, which are critical to managing with stress and trauma. Constructing them is necessary to maintain balance and the well-being, satisfaction, and quality of life of individuals and societies [49]. Chen and Bonanno [50] suggest that diagnosing the long-term patterns of COVID-19 effects on mental health is important to better understand risk factors and resilience. Such findings can provide valuable information that can be incorporated into prevention and intervention programs to help the general public cope with evolving multifaceted challenges at different stages of the pandemic [51]. Building resilience-based prevention programs can also activate other resources to overcome the acute effects of the pandemic, which will ultimately affect the mental health of individuals. The development of intervention programs can also help boost resilience and improve well-being among the affected populations. The results of the current study show important practical implications for improving mental health among people confronted with the COVID pandemic [52,53].

Previous literature on resilience theory and empirical findings indicate the importance of personality resources in promoting well-being and minimizing anxiety and stress in coping with the COVID-19 pandemic. Therefore, the analyzed problem is of key importance for researchers and clinicians, as identifying resilience resources and risk factors is important in order to better understand the functioning of individuals in the field of mental health. Such findings provide valuable information that can be incorporated into preventive and therapeutic interventions to help the general public cope with the evolving multifaceted challenges at different stages of the pandemic [49,50,51,52,53].

### Purpose of the of Study

Studies that have assessed the effects of COVID-19 from a mental health perspective commonly highlight negative indicators and the mental disorders it can lead to [54,55,56]. However, it has been argued that focusing on positive indicators that make up a healthy psychological perspective, such as resilience and well-being, is more effective in developing adequate mental health [51,57,58]. Therefore, this paper aimed to assess the state of resilience and well-being in the Polish population during the COVID-19 pandemic. This research perspective can provide important insights into how individuals can become mentally stronger during the pandemic. We also assessed the relationship between resilience and mental health. Finally, we tested the mediating role of COVID-19 anxiety, persistent thinking, and the stress burden in the relationship between mental health and resilience. Based on the literature review described above [39,40,41,42,43,44,45,46,47,48], we suppose that the relationship between resilience and well-being will be mediated by (H1) anxiety over coronavirus, (H2) obsessive thinking about COVID-19, and (H3) the stress burden. Due to these formulated hypotheses, we assume that including a mediator in the analysis will decrease the strength of the relation between the independent variable (resilience) and the dependent variable (well-being).

## 2. Method

### 2.1. Participants and Procedure

The study was conducted from July to December 2020 with the approval of the university ethics committee (#15/IV/2020). A total of 1758 participants (73% of whom were female) aged 18–80 years (*M* = 29.08; *SD* = 11.33) participated in the study. The invitation to participate in the study was distributed through social media (e.g., Facebook) and websites (news portals). The Google Forms platform was used to collect data. Each participant gave informed consent to participate in the study anonymously. The procedure consisted of completing a questionnaire to measure resilience, coronavirus anxiety, obsessive thinking, stress, and well-being. The questionnaire took approximately 30 min to complete. At the end, each participant declared their age and sex.

### 2.2. Measures

The World Health Organization’s Five Well-Being Index (WHO-5) was used to assess participants’ well-being [59]. This short scale avoids symptom-related or negative phrasing and measures well-being instead of the absence of distress. Representative items include “I have felt cheerful and in good spirits” and “My daily life has been filled with things that interest me”. Respondents assess how often they had the respective feelings within the last 2 weeks, ranging from 0 (no times) to 5 (all the time). Here, we report the sum scores, ranging from 0 to 25. A score <13 would indicate reduced well-being or even a depressive state. The Polish version showed satisfactory accuracy and internal consistency (*α* = 0.87) [60]. Sample items include “I have felt cheerful and in good spirits” and “I have felt calm and relaxed”.

Perceived stress burden (PSB). Perceived effects on daily life due to disease-related symptoms, feelings of being restricted in daily life by the COVID-19 pandemic, and feelings of being under pressure (i.e., stress and fear) due to the pandemic were measured using 5 items, ranging from 0 (not at all) to 100 (very strong). This stress’ scale is strongly related to reduced well-being and life satisfaction, but only marginally with awe/gratitude. These 5 items can be combined into a factor termed “perceived stress’’ with good internal consistency (*α* = 0.80) [61]. Sample items include “I feel anxiety and uncertainty” and “I’m feeling stressed”.

The Coronavirus Anxiety Scale (CAS) is a single-factor instrument designed to assess the severity of anxiety related to the psychological crisis caused by the new human coronavirus pandemic [62]. Participants respond to 5 statements (physical symptoms of anxiety) on a 5-point Likert scale, ranging from 0 (not at all) to 4 (almost every day). The CAS is a diagnostically accurate and reliable tool for assessing the severity of coronavirus anxiety. CAS scores correlated statistically significantly with general anxiety, depression, suicidal thinking, and drug and alcohol use. The Polish version showed satisfactory accuracy and internal consistency (*α* = 0.86) [42]. Sample items include “I lost interest in eating when I thought about or was exposed to information about the coronavirus” and “I felt nauseous or had stomach problems when I thought about or was exposed to information about the coronavirus”.

The Obsession with COVID-19 Scale (OCS) is a self-reported mental health screen for persistent and disturbed thinking about COVID-19 [63]. Participants express their attitude toward 4 statements on a 5-point Likert scale, ranging from 0 (not at all) to 4 (almost every day). The OCS is a reliable instrument with solid factorial (single-factor) and construct (correlated with coronavirus anxiety, spiritual crisis, alcohol/drug coping, extreme hopelessness, and suicidal ideation) validity. The diagnostic properties of the OCS (81 to 93% sensitivity and 73 to 76% specificity) are comparable to related screening instruments such as the General Health Questionnaire (GHQ). The Polish version of the OCS was characterized by satisfactory psychometric properties (*α* = 0.82) [43]. Sample items include “I had disturbing thoughts that I may have caught the coronavirus” and “I had disturbing thoughts that certain people I saw may have the coronavirus”.

The Brief Resilience Scale (BRS) is a reliable means of assessing resilience as the ability to bounce back or recover from stress [64]. The BRS has 6 items with a 5-point Likert response scale, ranging from 1 (strongly disagree) to 5 (strongly agree); 3 items are positively phrased and 3 are negatively phrased. The BRS is scored by reverse coding items 2, 4, and 6, and then calculating the mean of the 6 items. The BRS is reliable and was measured as a unitary construct. It was predictably related to personal characteristics, social relations, coping, and health in all samples. It was negatively related to anxiety, depression, negative affect, and physical symptoms when other resilience measures and optimism, social support, and type D personality were controlled. The Polish version of the BRS was characterized by good internal compatibility assessment based on Cronbach’s alpha (*α* = 0.88) and McDonald’s omega (*ω* = 0.88) [65]. Sample items include “I tend to bounce back quickly after hard times” and “I have a hard time making it through stressful events”.

### 2.3. Statistical Analysis

Data analysis was conducted in IBM SPSS Statistics 26 and the PROCESS macro in version 3.2 [66]. Pearson’s correlation analysis was used to determine the relations between variables. The significance level was determined at *p* < 0.050. The effect size was assessed based on R^2^. In order to verify the mediating role of COVID-19 anxiety, stress burden, and obsession with COVID-19 on the relationship between resilience and well-being, a bootstrapping analysis (for 2000 samplings) was carried out to establish 95% percentile confidence intervals for the estimated effects. When the value of the confidence interval exceeds 0, it means that the given effect is insignificant.

## 3. Results

The mean values obtained in the study, together with the standard deviation regarding the controlled variables of resilience (BRS), coronavirus anxiety (CAS), persistent thinking (OCS), perceived stress (PBS), and well-being (WHO-5), as well as correlation coefficient values, are depicted in Table 1. Statistically significant relations were observed between resilience and coronavirus anxiety, persistent thinking, perceived stress, and well-being; between coronavirus anxiety and persistent thinking, perceived stress, and well-being; between persistent thinking and perceived stress and well-being; and between perceived stress and well-being. In addition, a significant association was observed between age and resilience (*r* = 0.14; *p* < 0.010), perceived stress (*r* = −0.15; *p* < 0.010), and well-being (*r* = 0.13; *p* < 0.010). In this study, sex did not have a statistically significant impact on the controlled variables.

**Figure 1:**
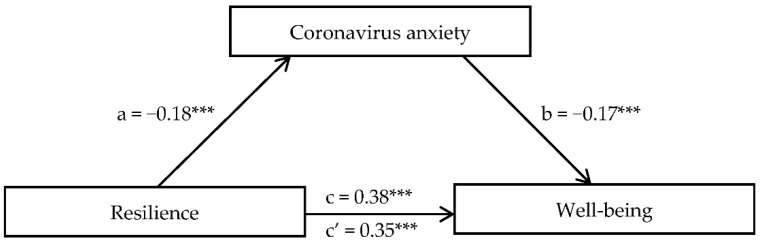
Coronavirus anxiety as mediator in relationship between resilience and well-being. (*** *p* ≤ 0.001).

Bootstrap sampling analysis (5000) with a 95% confidence interval displayed significant partial mediators for the relationship between resilience and well-being. An important mediator was coronavirus anxiety, persistent thinking, and perceived stress. The total effect (c path) amounted to *β* = 0.38 (*t* = 15.89, *p* < 0.001; *R*^2^ = 0.15). In the case of coronavirus anxiety, the regression coefficient of the independent variable on the mediator (a path) amounted to *β* = −0.18 (*t* = −7.85, *p* < 0.001; *R*^2^ = 0.03). The mediator regression coefficient on the dependent variable with simultaneous control of the independent variable (b path) amounted to *β* = −0.17 (*t* = −7.54, *p* < 0.001; *R*^2^ for the entire model = 0.17). Mediation decreased the strength of the relationship between resilience and mental well-being in a direct effect (c′ path), amounting to *β* = 0.35 (*t* = 15.89, *p* < 0.001). The a path for persistent thinking totaled *β* = −0.17 (*t* = −7.28, *p* < 0.001; *R*^2^ = 0.03), the b path totaled *β* = −0.25 (*t* = −11.79, *p* < 0.001; *R*^2^ for the entire model = 0.21), and the c′ path totaling *β* = 0.34 (*t* = 15.69, *p* < 0.001). The a path for perceived stress totaled *β* = −0.29 (*t* = −12.82, *p* < 0.001; *R*^2^ = 0.09), the b path totaled *β* = −0.41 (*t* = −19.45, *p* < 0.001; *R*^2^ for the entire model = 0.30), and the c′ path totaled *β* = 0.26 (*t* = 12.55, *p* < 0.001). The significance level for the effects is presented in Table 2. To illustrate the mediation pattern, Figure 1 shows the relationship of resilience and well-being with coronavirus anxiety as a mediator.

## 4. Discussion

The main goal of this paper was to investigate potential mental health mediators in the relationship between resilience and well-being. Although early studies looked at this relationship [67,68,69], there is a lack of studies assessing the mediating role of anxiety, aggravating stress, and persistent thinking in connection with the pandemic. The first finding shows that anxiety over COVID-19 partially mediates the relationship between resilience and well-being, which supports our first hypothesis (H1). The second hypothesis (H2) was also confirmed. Obsessive thinking about COVID-19 partially mediates the relationship between resilience and well-being. The third hypothesis (H3), that perceived stress in a pandemic might mediate the relationship between resilience and well-being, is also confirmed. For this reason, by preventing the development of COVID-19 anxiety, obsessive thoughts, and burdensome stress, resilience helps protect well-being. Therefore, it should be said that building resilience helps in coping with stress, persistent thoughts, and anxiety caused by COVID-19. In other words, resilience minimizes the anxiety, obsessive thinking, and burdensome stress of a pandemic that disrupt daily functioning. The obtained data seem to confirm the previous findings, which indicated that resilience is a helpful resource in protecting mental health. The positive psychological approach assumes that resilience is a human strength and that instead of treating mental disorders, developing psychological strength is an alternative way to protect mental health [51,57,58,70]. So far, it has been shown that intervention programs focused on positive psychology reduce, inter alia, stress and anxiety and increase aspects of health [71,72,73]. Taking the above into account, our research findings are consistent with previous theoretical knowledge in the field of positive psychology, as well as with the results of experimental and other cross-sectional studies [47,48,74,75].

We found resilience to be a wellness-enhancing construct that may have temporal or long-term psychological consequences, and tested the mediating effects of anxiety, stress, and obsessive thinking on the relationship between resilience and well-being. Although mediation studies do not directly test causality, they are very fruitful in terms of providing data that help change the therapeutic or interventional approach [76]. Our report is an important contribution to the scope of research to date on health-related psychological factors, taking into account positive and negative indicators in the general population. Through general population research, this study complements previous research in which the effects of COVID-19 have generally been studied among healthcare professionals [77].

This study also looked at the relationship between resilience and mental health. Resilience was moderately positively associated with well-being and negatively and weakly associated with anxiety, obsessive thinking, and perceived stress. Our research results confirm earlier research findings [35,49,54,78,79,80], which indicate a positive role of resilience in terms of well-being and buffering in terms of negative health aspects, such as perceived stress, anxiety, or persistent thinking. Moreover, a significant positive relationship was observed between age and resilience as well as age and well-being. Age was also negatively related to perceived stress, which corresponds to previous findings.

It is worth noting that the average results in terms of resilience were comparable to the Polish adaptation studies conducted by Konaszewski and colleagues [65]. Although research so far has shown a lower level of resilience during the COVID-19 pandemic, our data were collected during the period of relaxed government restrictions. It was also influenced by a decrease in infections, hospitalizations, and deaths. Moving within the country has become permissible, as well as physical exercise outside, praying in small groups, and meeting with loved ones. These factors, according to PeConga and colleagues [37,38], can moderate the level of resilience during a pandemic. In the studied population, the resilience result can be described as average. On the other hand, the result in terms of well-being shows a relatively low level. Scores below 13 indicate depressed mood or even a depressive state [61]. Poor mental well-being can make it difficult for individuals to use psychological resources to deal with their burdens [81]. The intensity of perceived stress indicates a feeling of low burden in the studied population [61]. The average level of anxiety about the coronavirus and persistent thinking about COVID-19 was within the fourth sten score, which proves the low intensity of these phenomena among the study participants. The obtained data correspond to previous research in this area, as high intensity of these variables is mainly observed in clinical groups [42,43].

## 5. Implications

In terms of practical implications, our findings offer important guidance for the development of resilience-based interventions to protect the mental health of individuals, improve their quality of life, and provide policy recommendations. The results of this study essentially indicate that developing strong psychological resources can help protect people’s mental health against the anxiety, persistent thinking, and stress caused by the spread of an infectious disease. Resources such as resilience can be enhanced through psychological intervention and therapeutic programs [82,83,84,85]. Protecting mental health with regard to the risk of infection is key to the successful fight against the pandemic. The purpose of such interventions may include the following: (a) They may help in maintaining mental health so that individuals can function with the provision of primary health care and health services in times of crisis without psychological problems. (b) They may help in identifying individuals who may be vulnerable to stressors due to their inability to cope with adversity during the pandemic. In addition to resilience-enhancing interventions, medical clinics can be effectively used to provide services to individuals who develop symptoms of mental health problems such as anxiety, obsessive thinking, and stress disorder disorders during a pandemic [50,51,52,53,54,55,56,57,58,59,60,61,62,63]. (c) Targeted psychological interventions for communities affected by the spread of an infectious disease and special support for people at high risk of mental illness are recommended, by improving the diagnosis of mental disorders and access to such interventions (especially those provided online, which reduces the risk of infection), which may ultimately help to improve people’s wellbeing.

## 6. Limitations

This study has some limitations. First, while questionnaire assessment is an important method of measuring mental health problems and related factors, relying solely on self-reports may run the risk of encountering a common method error. It is suggested that future research use alternative methods to complement the current findings. Second, given the cross-sectional nature of the study design, it was difficult to draw conclusions about the cause-and-effect relationship between the variables studied. Therefore, longitudinal studies are necessary to confirm the cause-and-effect sequence of relationships between the studied variables. Such data are particularly relevant to the design of mental health programs in the wake of a pandemic. In addition, the study did not assess what percentage of respondents contracted coronavirus or had a sick/deceased relative. The severity of the variables in these groups may differ from the data collected from participants in the general population. Finally, the current findings should be replicated in other clinical and non-clinical trials. Despite these limitations, these results may shed light on a possible underlying mechanism between resilience and well-being by considering the mediating role of stress, obsessive thinking, and anxiety. The results may also point to the direction of future research to investigate the relationship between resilience and mental health in terms of both negative and positive indicators, and provide opportunities for implementing resilience-based programs.

## 7. Conclusions

Protecting people’s mental health from the consequences of COVID-19 is currently one of the most psychologically and socially challenging issues. The results of the present study clearly indicate that resilience as a protective factor is associated with reduced anxiety over COVID-19, perceived stress burden, obsessive thoughts about the pandemic, and increased well-being of individuals. In other words, resilience plays an important role in minimizing negative and enhancing positive health indicators in the face of challenging life events. Thus, it is worthwhile to develop individuals’ psychological resources by strengthening emotional, cognitive, mental, physical, and spiritual resilience, among other aspects. Training in these areas can improve resilience and well-being and reduce stress and anxiety by teaching individuals to view life’s inevitable challenges as opportunities.

## Figures and Tables

**Table 1:** Descriptive statistics and correlations (*n* = 1758).

	M (SD)	Min	Max	1.	2.	3.	4.
1. Resilience	18.64 (5.28)	6	30	1			
2. Coronavirus anxiety	1.59 (2.95)	0	20	−0.18 ***	1		
3. Persistent thinking	3.03 (3.18)	0	16	−0.17 ***	0.54 ***	1	
4. Perceived stress	173.03 (123.69)	0	500	−0.29 ***	0.36 ***	0.44 ***	1
5. Well-being	13.72 (5.72)	0	25	0.38 ***	−0.23 ***	−0.31 ***	−0.48 ***

*** *p* < 0.001.

**Table 2:** Mediating role of coronavirus anxiety, persistent thinking, and perceived stress in relationship between resilience and well-being (*n* = 1758).

	a Path	b Path	c Path	c′ Path	Indirect Effect and B (SE)	95% CI Lower Upper
Resilience → Coronavirus anxiety → Well-being	−0.18 ***	−0.17 ***	0.38 ***	0.35 ***	0.031 (0.006)	0.020; 0.043
Resilience → Persistent thinking → Well-being	−0.17 ***	−0.25 ***	0.38 ***	0.34 ***	0.044 (0.007)	0.030; 0.058
Resilience → Perceived stress → Well-being	−0.29 ***	−0.41 ***	0.38 ***	0.26 ***	0.119 (0.010)	0.099; 0.141

*** *p* < 0.001; a path effect of independent variable on mediator; b path effect of mediator on dependent variable; c path effect of independent variable on dependent variable; c′ path direct effect of independent variable on dependent variable while controlling for mediator.

## Data Availability

The data presented in this study are available on request from the corresponding author.
